# Association of Vitamin D, Zinc and Selenium Related Genetic Variants With COVID-19 Disease Severity

**DOI:** 10.3389/fnut.2021.689419

**Published:** 2021-06-04

**Authors:** Nikola Kotur, Anita Skakic, Kristel Klaassen, Vladimir Gasic, Branka Zukic, Vesna Skodric-Trifunovic, Mihailo Stjepanovic, Zorica Zivkovic, Olivera Ostojic, Goran Stevanovic, Lidija Lavadinovic, Sonja Pavlovic, Biljana Stankovic

**Affiliations:** ^1^Laboratory for Molecular Biomedicine, Institute of Molecular Genetics and Genetic Engineering, University of Belgrade, Belgrade, Serbia; ^2^Clinic of Pulmonology, Clinical Center of Serbia, Belgrade, Serbia; ^3^Medical Faculty, University of Belgrade, Belgrade, Serbia; ^4^Children's Hospital for Lung Diseases and Tbc, MC Dr Dragisa Misovic, Belgrade, Serbia; ^5^Faculty of Pharmacy Novi Sad, Business Academy, Novi Sad, Serbia; ^6^Clinic for Infectious and Tropical Diseases, Clinical Centre of Serbia, Belgrade, Serbia

**Keywords:** DHCR7, CYP2R1, nutrigenetics, COVID-19, population genetics

## Abstract

**Background:** COVID-19 pandemic has proved to be an unrelenting health threat for more than a year now. The emerging amount of data indicates that vitamin D, zinc and selenium could be important for clinical presentation of COVID-19. Here, we investigated association of genetic variants related to the altered level and bioavailability of vitamin D, zinc and selenium with clinical severity of COVID-19.

**Methods:** We analyzed variants in genes significant for the status of vitamin D (*DHCR7*/*NADSYN1* rs12785878, GC rs2282679, *CYP2R1* rs10741657, and *VDR* rs2228570), zinc (*PPCDC* rs2120019) and selenium (*DMGDH* rs17823744) in 120 Serbian adult and pediatric COVID-19 patients using allelic discrimination. Furthermore, we carried out comparative population genetic analysis among European and other worldwide populations to investigate variation in allelic frequencies of selected variants.

**Results:** Study showed that *DHCR7/NADSYN* rs12785878 and *CYP2R1* rs10741657 variants were associated with severe COVID-19 in adults (*p* = 0.03, *p* = 0.017, respectively); carriers of *DHCR7/NADSYN* TG+GG and *CYP2R1* GG genotypes had 0.21 and 5.9 the odds for developing severe disease, OR 0.21 (0.05–0.9) and OR 5.9 (1.4–25.2), respectively. There were no associations between selected genetic variants and disease severity in pediatric patients. Comparative population genetic analysis revealed that Serbian population had the lowest frequency of *CYP2R1* rs10741657 G allele compared to other non-Finish Europeans (0.58 compared to 0.69 and 0.66 in Spanish and Italian population, respectively), suggesting that other populations should also investigate the relationship of *CYP2R1* variant and the COVID-19 disease course.

**Conclusion:** The results of the study indicated that vitamin D related genetic variants were implicated in severe COVID-19 in adults. This could direct prevention strategies based on population specific nutrigenetic profiles.

## Introduction

COVID-19 pandemic has proved to be an unrelenting health threat for more than a year now. More contagious and more deadly SARS-CoV-2 variants are a major concern, especially because effective, causal therapy is still unavailable, and vaccination coverage rates are lower than anticipated. Moreover, protection from vaccination is likely to last only for a season or two. One of the strategies to save lives is to identify groups at risk of severe COVID-19 disease and to implement measures aimed at those groups. The elderly, male, obese, chronic disease patients, patients with malignancies, immunocompromised, dark-skinned, socioeconomically disadvantaged and tobacco users suffered the most death toll from COVID-19 (1, 2). Also, interindividual genetic variations might be implicated in more severe COVID-19 (3, 4).

Another strategy directed toward reducing severe COVID-19 symptoms relies on high dose vitamins and trace elements (micronutrients) supplementation. Among micronutrients important for adequate immune function, vitamin D, zinc and selenium are of particular importance for coping with viral, respiratory infections, such as COVID-19 (5). Adequate status of these micronutrients is not only important for immune function and viral clearance, but also might mitigate life-threatening complications of SARS-CoV-2 infection, such as thrombosis and uncontrolled inflammation which leads to cytokine storm. Namely, vitamin D via vitamin D receptor (VDR) mediates immune function and regulation, strengthening of epithelial barriers and antioxidant defense, and also regulates expression of SARS-CoV-2 receptor, ACE2 (6–8). Zinc exerts direct anti-viral effects and also serves as a cofactor of dozens of proteins important for immune function and regulation, and antioxidative defense (9). Selenium via selenoproteins regulates immune function and provides antioxidant defense and vaso-protection (10).

Although important for various processes in our body, a lot of people worldwide have inadequate status of vitamin D, zinc or selenium. There is a pandemic of vitamin D deficiency, especially in higher latitudes due to inadequate sun exposure necessary for the synthesis of this vitamin (11). Selenium deficiency is prevalent in Europe and other parts of the world where soil is poor of this element, while zinc status is compromised predominantly in underdeveloped regions (12). Vitamin D, zinc and selenium deficiency is associated with factors of COVID-19 severity, such as old age, obesity, diabetes, dyslipidemia and chronic and acute inflammation. COVID-19 patients have lower status of vitamin D, zinc and selenium than healthy individuals (13). Also, patients with severe COVID-19 disease have lower status of vitamin D, zinc and selenium than mild and moderate disease patients (14–16). Preliminary evidence from intervention studies suggests that supplementation with these micronutrients protects from severe COVID-19 disease (17, 18).

Apart from chronic and acute health conditions, lifestyle and nutrition, genetic factors also play a role in bioavailability and status of vitamin D, zinc and selenium. Several large genome-wide association studies (GWAS) identified variants located near genes involved in synthesis, transport and metabolism of vitamin D, namely *DHCR7/NADSYN1, GC* and *CYP2R1*, respectively (19–21). Variants in these genes are associated with serum level of 25OHD, which is a measure of vitamin D status. Also, genetic variants in *VDR* gene, which codes for vitamin D receptor that regulates expression of more than 200 human genes (22), have been shown to increase susceptibility to acute lower respiratory infections in children, as well as several inflammatory and autoimmune diseases (23–26). GWAS studies comprising three large independent cohorts associated selenium status measured in whole blood and erythrocytes (27), as well as nails (reflects longer duration of selenium exposure) (28) with a locus near *DMGDH* gene. The same locus was implicated in a response to selenium supplementation in another GWAS (29). Zinc nutrigenetics is less studied, but a few candidate variants associated with status of this trace elements are identified in a large GWAS, and the most significant association was noted for rs2120019 variant near *PPCDC* gene (27).

Building on this premise, we hypothesize that genetic variants linked to suboptimal level or decreased bioavailability of vitamin D, zinc and selenium, may also serve as markers of disease course in COVID-19 patients, influenced by the level of these micronutrients. Therefore, we evaluated genetic determinants of vitamin D (*DHCR7/NADSYN1* rs12785878, *GC* rs2282679, *CYP2R1* rs10741657, *VDR* rs2228570), zinc (*PPCDC* rs2120019) and selenium (*DMGDH* rs17823744) as risk factors for severe forms of COVID-19. Furthermore, we attempted to investigate the inter-population diversity of the selected genetic variants. Inter-population nutrigenetic variability could suggest different relevance of nutrigenetic factors in populations exposed to different nutritional habits and specific environmental factors. These data should be taken into account when evaluating the possible relationship of nutrigenetic variants with severe COVID-19 forms in other populations struck by the pandemic.

## Materials and Methods

### Patients

Present study included 120 patients with diagnosis of COVID-19 (adult and pediatric) who were treated in tertiary healthcare institutions, the Clinic of Pulmonology, Clinical Center of Serbia and Children's Hospital for Lung Diseases and Tuberculosis, Medical Center “Dr Dragiša Mišović,” Belgrade, Serbia, between April and June of 2020. For all patients, a COVID-19 infection was defined as a positive SARS-CoV-2 laboratory test (viral RNA RT-PCR test from nasopharyngeal swabs). From each patient, peripheral blood sample was collected for DNA isolation and further genetic analyses.

Clinical data were assessed using electronic health records. From a total of 120 COVID-19 patients included in the study, clinical data were available for 115 patients, 73 adults and 42 pediatric.

Adult COVID-19 patients were categorized into mild, moderate or severe clinical course according to the National Institutes of Health (NIH) Definition of COVIID-19 Disease Severity (COVID-19 Treatment Guidelines Panel. *Coronavirus Disease 2019 (COVID-19) Treatment Guidelines*. 2020. Available at: http://www.covid19treatmentguidelines.nih.gov), as follows: *mild* – patients with symptoms such as fever, fatigue, cough, myalgia, and headache, but without dyspnea or pneumonia; *moderate*- those with evidence of pneumonia based on imaging showing up to 50% of lung involvement, who had blood oxygen saturation >93% on room air; *severe*- patients who demonstrated pneumonia with >50% of lung involvement on imaging or had blood oxygen saturation level equal to or <93% on room air and required supportive oxygen therapy.

Pediatric patients were enrolled during the first peak of the COVID-19. At that time, SARS-CoV-2 positive pediatric patients were admitted to the hospital if they had COVID-19 symptoms or for observation due to previous health issues. Most of the recruited pediatric patients (90.5%) demonstrated mild form of the disease. Therefore, for the purpose of this study, we classified pediatric patients into 2 groups – *symptomatic*, who were diagnosed with pneumonia or any typical COVID-19 symptoms such as cough, fever, sore throat, fatigue, and headache or *asymptomatic*, patients tested positive for SARS-CoV-2 with medical history of recurrent respiratory diseases.

This study was approved by the Ethics Committee of the Institute of Molecular Genetics and Genetic Engineering University of Belgrade (approval for sample collection and biobank formation O-EO-016/2020, 06.05.2020.; approval for the genetic study O-EO-016/2020/1, 03.09.2020). Informed consent was obtained from each participant or their parents/legal guardians. The study was performed in accordance with the Declaration of Helsinki.

### Genotyping

Genomic DNA was isolated from the whole blood samples using QIAamp DNA Blood Mini Kit (Qiagen) and stored at −20°C until analysis.

Genetic variants *DHCR7/NADSYN1* rs12785878, *GC* rs2282679, *CYP2R1* rs10741657, *VDR* rs2228570, *PPCDC* rs2120019 and *DMGDH* rs17823744 were analyzed using TaqMan SNP Genotyping Assays (Thermo Fisher Scientific) (C__32063037_10, C__26407519_10, C__2958430_10, C__12060045_20, C__304992_10, C__32728760_10), according to the manufacturer's instructions with modifications. A total volume of 8 μl PCR reaction contained 36 ng of DNA, 1x Taqman Genotyping Master Mix and 1x TaqMan SNP Genotyping Assays (Thermo Fisher Scientific). Genotyping was performed on a real-time PCR system (Applied Biosystems 7500, Thermo Fisher Scientific). Genotype calling was implemented with built-in 7500 System Software v1.3.1 (Thermo Fisher Scientific).

### Statistical Analysis

Statistical analyses were performed in software SPSS v23.0 and R v.3.6.2.

Differences in demographic and clinical data between COVID-19 mild/moderate/severe subgroups were examined using adequate statistical tests for continuous and discrete data (Mann-Whitney U test, Chi-square or Fisher exact test).

For genetic data, Hardy-Weinberg equilibrium was examined to assess the performance of the genotyping assays. Differences in genotype and allele counts distribution for each variant between COVID-19 subgroups were tested using the Chi-squared test. Impact of each genetic variable on severity of the COVID-19 was estimated by odds ratio with 95% confidence interval using a logistic regression model in order to control for confounding factors, such as age and gender.

Frequencies of the analyzed variants were compared between group of Serbian COVID-19 patients and other populations using two openly available variant databases, 1,000 Genomes Project (1KGP) and Genome Aggregation Database (gnomAD) v.3.0 (30, 31). Chi square or Fisher exact tests were used to measure significant differences in allelic distributions between Serbian and European populations (Finish and non-Finish, particularly Italian, Spanish, Britain and USA with European ancestry), and other worldwide populations (African, East and South Asian, American Latino/Admixture and Ashkenazi Jewish), applying Bonferroni correction for multiple testing.

We examined the level of population genetic variability at each genetic loci using the maximal global difference in risk allele frequency (delta AF), which was calculated by subtracting the maximum and the minimum allele frequency across analyzed populations.

All tests were bi-directional. The threshold for statistical significance was defined using appropriate multiple comparisons correction methods, such as Bonferroni or Benjamini-Hochberg false discovery rate (FDR).

## Results

### Description of COVID-19 Patient Groups

In the selected study group, 73 were adults while 42 were pediatric patients. Among adults 65.8% were females and the median age was 43 (range 20–82); among pediatric patients 38.1% were females and median age was 9 (range 0.3–17). Demographic and clinical descriptions of COVID-19 patients are presented in Table 1.

**Table 1:** Demographic and clinical characteristic of COVID-19 patients.

	**Adult COVID-19 (*****n*** **=** **73)**			
	**Mild**	**Moderate**	**Severe**	***p***
N (%)	35 (47.9)	21 (28.8)	17 (23.3)	
Age, median [IQR]	37 [29–49]	41 [33.7–52]	61 [49.3–67]	** <0.001**
Gender, Female n (%)	28 (80)	11 (52.4)	9 (52.9)	0.05
Obesity, n/available (%)	5/33 (14)	7/19 (36.8)	6/16 (37.5)	0.1
Hypertension, n/available (%)	7/35 (20)	5/21 (23.8)	10/16 (62.5)	0.01
Diabetes, n/available (%)	1/35 (3)	2/21 (9.5)	4/16 (25)	0.05
ACE inhibitors, n/available (%)	5/35 (14.3)	2/21 (9.5)	8/15 (53.3)	**0.006**
% SatO_2_, median [IQR]	98 [98–99]	97.5 [97–99]	95.5 [87–97]	** <0.001**
CRP, median [IQR]	0.6 [0.2–4.3]	17.2 [4.7–91.7]	135.6 [96.7–260.4]	** <0.001**
Febrile, n/available (%)	7/35 (20)	11/21 (52.4)	14/16 (87.5)	** <0.001**
Lymphopenia (<0.8 x109/L), n/available (%)	7/34 (20.6)	9/21 (42.9)	15/16 (93.8)	** <0.001**
Thrombocytopenia (<150,000/mm^3^), n/available (%)	5/33 (15.2)	3/21 (14.3)	9/16 (56.3)	**0.005**
	**Pediatric COVID-19 (*****n*** **=** **42)**			
	**Asymptomatic**	**Symptomatic**	***p***	
N (%)	20 (47.6)	22 (52.4)		
Age, median [IQR]	9.0 [7.3–13.5]	10.0 [1.7–15.0]	0.9	
Gender, Female %	8/20 [40]	8/22 (36.4)	0.8	
Obesity, n/available (%)	0/20 (0)	1/22 (4.5)	1	
Hypertension, n/available (%)	0/20 (0)	1/22 (4.5)	1	
Diabetes, n/available (%)	0/20 (0)	0/22 (0)	NA	
% SatO_2_	98.0 [97.0–99.0]	98.0 [96.7–98.3]	0.3	
CRP, median [IQR]	0.45 [0.2–0.97]	2.7 [0.53–11.3]	**0.002**	
Lymphopenia (<0.8 x109/L), n/available (%)	0/20 (0)	4/22 (18.2)	0.1	
Thrombocytopenia (<150,000/mm^3^), n/available (%)	0/20 (0)	0/22 (0)	NA	

*To deal with missing data, each variable count was presented along with the total available data for that category (n/available). Differences between groups were tested using Mann-Whitney U test for continuous data, or Chi-square/Fisher exact test for discrete data. Benjamini-Hochberg correction for multiple testing was applied (FDR of 0.05); significant probabilities after correction were presented in bold. IQR, interquartile range; SatO_2_, Blood oxygen saturation; CRP, C-reactive protein*.

In the group of adult COVID-19 patients, 10 (13.7%) required supportive oxygen therapy and 3 (4%) had COVID-19 related death outcomes. Examining available clinical data, patients were categorized into mild (*n* = 35), moderate (*n* = 21) or severe (*n* = 17) disease groups. Age distribution of the patients significantly varied between groups; gradual increase in participants' age has been observed across mild, moderate and severe groups (*p* < 0.001). In the severe group, a significantly higher number of patients suffered from hypertension, diabetes and were using ACE inhibitors, compared to mild and moderate groups (*p* < 0.001, *p* < 0.001, *p* < 0.001, respectively). The highest percent of lymphopenia and thrombocytopenia events were observed in the severe group, 93.8 and 56.3%, respectively. Also, patients with severe COVID-19 had higher levels of CRP, compared to patients with moderate and mild disease (*p* < 0.001).

Pediatric COVD-19 patients were categorized into two groups – asymptomatic (*n* = 20) and symptomatic (*n* = 22). There was no significant difference in distribution of age, gender, nor in the presence of comorbidity between the two groups, except past respiratory diseases. The level of CRP was significantly higher in COVID-19 symptomatic compared to asymptomatic pediatric patients (*p* = 0.003).

### Association of Genetic Variants With the Risk of Severe COVID-19

COVID-19 patients were genotyped for variants in *DHCR7/NADSYN1, GC, CYP2R1, VDR, PPCDC* and *DMGDH* genes (Table 2). Distributions of genotypes of all analyzed variants were in accordance with Hardy-Weinberg equilibrium.

**Table 2:** Genotype and allele frequencies of COVID-19 patients (*n* = 120).

**Genetic variant**	**Genotype**	***n* (freq)**	**HW**	**Allele**	**freq**
DHCR7/NADSYN1 rs12785878	TT	56 (0.47)	0.2	T	0.70
	TG	56 (0.47)		G	0.30
	GG	8 (0.07)			
GC rs2282679	TT	59 (0.50)	0.8	T	0.70
	TG	49 (0.41)		G	0.30
	GG	11 (0.09)			
CYP2R1 rs10741657	GG	36 (0.3)	0.1	G	0.58
	GA	67 (0.56)		A	0.42
	AA	17 (0.14)			
VDR rs2228570	GG	53 (0.44)	0.5	G	0.65
	GA	51 (0.43)		A	0.35
	AA	16 (0.13)			
PPCDC rs2120019	TT	65 (0.54)	0.4	T	0.75
	TC	49 (0.41)		C	0.25
	CC	6 (0.05)			
DMGDH rs17823744	AA	87 (0.72)	0.7	A	0.85
	AG	31 (0.26)		G	0.15
	GG	2 (0.02)			

*HW, hardy weinberg equilibrium; freq, frequency (number of observations/total number)*.

We compared the distribution of analyzed genotypes between different COVID-19 severity groups. In the group of adult patients, two outcomes were considered in the logistic regression model – first included both mild and moderate disease, and second was severe disease (Table 3). Logistic regression used genotypes of analyzed variants as variables, controlled for age and gender in multivariate models. To increase the statistical power of the study, a dominant genetic model has been applied, in particular, a group of homozygous carriers of more frequent allele were compared to heterozygous and homozygous carriers of the less frequent, minor allele. Analysis showed that carriers of *DHCR7/NADSYN1* G allele (TG+GG), associated with lower levels of 25OHD were 4.9 times less likely (or had 0.21 the odds) to develop a severe form of COVID-19, compared with carriers of TT genotype. When adjusted for age and gender, observed association was still significant (OR 0.21, 95% CI 0.05–0.9, *p* = 0.03). Association has also been observed between severe COVID-19 and *CYP2R1* GG genotype, previously related to decreased levels of 25OHD. In the logistic regression model, *CYP2R1* GG carriers had 5.9 times higher odds to develop severe disease, adjusted for age and gender (OR 5.9, 95% CI 1.4–25.2, *p* = 0.017). Additionally, we applied adjustment for obesity in multivariate logistic regression models, showing that *DHCR7/NADSYN1* rs12785878 and *CYP2R1* rs10741657 variants remained significant predictors of severe COVID-19 in adults (OR 0.22, 95% CI 0.06-0.93, *p*= 0.02; OR 4.9, 95% CI 1.5-16.4, *p*= 0.009, respectively). Our study did not show association of *GC* and *VDR* variants with higher risk of severe COVID-19 in adults.

**Table 3:** Association of the analyzed genotypes with the risk of severe COVID-19 disease in adults.

		**Adult COVID-19 (*****n*** **=** **73)**			**Mild+moderate vs. severe disease**				
**Genetic variant**	**Genotype**	**Mild (*n* = 35)**	**Moderate (*n* = 21)**	**Severe (*n* = 17)**	Genetic model^*^	**OR [CI]**	***P***	**OR^**adj**^ [CI]**	**p^**adj**^**
DHCR7/NADSYN1 rs12785878	TT	12 (34.3%)	9 (42.8%)	12 (70.6%)	TT^R^ vs. TG+GG	**0.25 [0.08–0.81]**	**0.02**	**0.21 [0.05–0.9]**	**0.03**
	TG	20 (57.1%)	9 (42.8%)	5 (29.4%)					
	GG	3 (8.6%)	3 (14.3%)	0 (0%)					
GC rs2282679	TT	16 (45.7%)	8 (40.0%)	8 (47.1%)	TT^R^ vs. TG+GG	0.8 [0.3–2.6]	0.8	1.3 [0.3–5.0]	0.7
	TG	17 (48.6%)	9 (45.0%)	8 (47.1%)					
	GG	2 (5.7%)	3 (15.0%)	1 (5.8%)					
CYP2R1 rs10741657	GG	11 (31.4%)	4 (19.0%)	10 (58.8%)	GA+AA^R^ vs. GG	**3.9 [1.3–12.1]**	**0.018**	**5.9 [1.4–25.2]**	**0.017**
	GA	18 (51.4%)	14 (66.7%)	4 (23.5%)					
	AA	6 (17.1%)	3 (14.3%)	3 (17.6%)					
VDR rs2228570	GG	15 (42.9%)	8 (38.1%)	7 (41.2%)	GG^R^ vs. GA+AA	1.0 [0.3–3.0)	1	0.8 [0.2–3.2]	0.7
	GA	15 (42.9%)	10 (47.6%)	8 (47.1%)					
	AA	5 (14.3%)	3 (14.3%)	2 (11.7%)					
PPCDC rs2120019	TT	20 (57.1%)	11 (52.4%)	7 (41.2%)	TT^R^ vs. TC+CC	1.8 [0.6–5.3]	0.3	1.4 [0.4–5.2]	0.6
	TC	14 (40.0%)	9 (42.8%)	8 (47.0%)					
	CC	1 (2.9%)	1 (4.8%)	2 (11.8)					
DMGDH rs17823744	AA	28 (80.0%)	17 (81.0%)	9 (53.0%)	AG+GG^R^ vs. AA	0.27 [0.09–0.9]	0.03	0.4 [0.1–1.8]	0.2
	AG	7 (20.0%)	2 (9.5%)	8 (47.0%)					
	GG	0 (0%)	2 (9.5%)	0 (0%)					

* *To increase statistical power of the study, a dominant genetic model was applied. Homozygous carriers of more frequent allele were compared to heterozygous and homozygous carriers of less frequent, minor allele. ^R^Group that included homozygous carriers of allele associated with higher levels of analyzed micronutrient was set to be a referent group in the logistic regression model*.

*OR, odds ratio; CI, confidence interval; adj, adjusted for age and gender. Bolded p-values remained significant after Benjamini-Hochberg correction for multiple testing (p <0.033), if FDR was set to 0.1*.

Homozygous carriers of *DMGDH* A allele, which has been related to lower levels of selenium, in our study were less likely to present severe form of COVID-19 (OR 0.2, 95% CI 0.09–0.9, *p* = 0.03). However, this association was not significant after correction for age and gender. Regarding zinc-related *PPCDC* variant, we observed no significant association with severe COVID-19 outcome in adult patients.

In pediatric COVID-19 cases, we applied a logistic regression model to predict two outcomes – asymptomatic and symptomatic disease, using genotype data (Table 4). No significant associations with increased risk for symptomatic pediatric COVID-19 disease were found with any of the analyzed genetic variants.

**Table 4:** Association of the analyzed genotypes with the risk of symptomatic COVID-19 disease in children.

		**Pediatric COVID-19 (*****n*** **=** **42)**		**Asymptomatic vs. symptomatic disease**				
**Enetic variant**	**Genotype**	**Asymptomatic (*n* = 20)**	**Symptomatic (*n* = 22)**	Genetic model^*^	**OR [CI]**	***p***	**OR^**adj**^ [CI]**	***p*^**adj**^**
DHCR7/NADSYN1 rs12785878	TT	11 (55%)	7 (31.8%)	TT^R^ vs. TG+GG	2.6 [0.7–9.2]	0.1	3.1 [0.8–11.8]	0.1
	TG	8 (40.0%)	14 (63.6%)					
	GG	1 (5.0%)	1 (4.5%)					
GC rs2282679	TT	10 (50.0%)	13 (59.1%)	TT^R^ vs. TG+GG	0.7 [0.2–2.3]	0.6	0.7 [0.2–2.5]	0.6
	TG	6 (30.0%)	8 (36.4%)					
	GG	4 (20.0%)	1 (4.5%)					
CYP2R1 rs10741657	GG	4 (20.0%)	5 (22.7%)	GA+AA^R^ vs. GG	1.2 [0.3–5.2]	0.8	1.2 [0.3–5.2]	0.8
	GA	13 (65.0%)	15 (68.2%)					
	AA	3 (15.0%)	2 (9.1%)					
VDR rs2228570	GG	9 (45.0%)	11 (50.0%)	GG^R^ vs. GA+AA	0.8 [0.2–2.7]	0.7	0.8 [0.2–2.8]	0.7
	GA	8 (40.0%)	8 (36.4%)					
	AA	3 (15.0%)	3 (13.6%)					
PPCDC rs2120019	TT	14 (70.0%)	11 (50.0%)	TT^R^ vs. TC+CC	2.3 [0.7–8.3]	0.2	2.6 [0.7–9.9]	0.2
	TC	6 (30.0%)	9 (40.9%)					
	CC	0 (0%)	2 (9.1%)					
DMGDH rs17823744	AA	14 (70.0%)	17 (77.3%)	AG+GG^R^ vs. AA	1.5 [0.4–5.8]	0.6	1.5 [0.4–5.8]	0.6
	AG	6 (30.0%)	5 (22.7%)					
	GG	0 (0%)	0 (0%)					

* *To increase statistical power of the study, a dominant genetic model was applied. Homozygous carriers of more frequent allele were compared to heterozygous and homozygous carriers of less frequent, minor allele. ^R^Group that included homozygous carriers of allele associated with higher levels of analyzed micronutrient was set to be a referent group in the logistic regression model. OR, odds ratio; CI, confidence interval; adj, adjusted for age and gender*.

### Comparative Population Genetics

**Figure 1:**
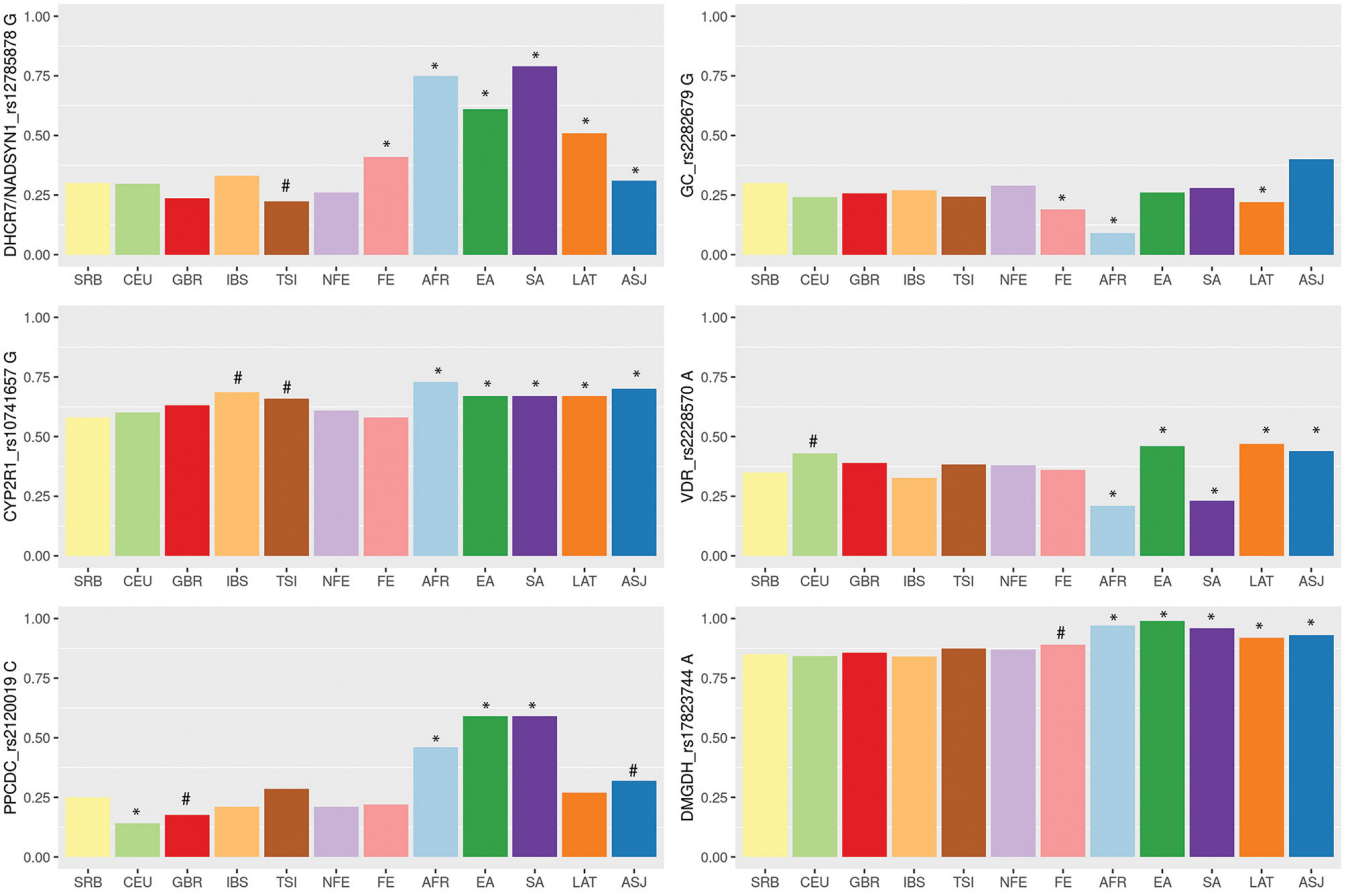
Variability of vitamin D, zinc and selenium related genetic loci among wordwide populations based on the 1,000 Genome project (1KGP), Genome Aggregation Database (gnomAD) and Serbian COVID-19 study group data. Populations: SRB, Serbian; CEU, Utah residents with Northern and Western European ancestry (1KGP); GBR, British in England and Scotland (1KGP); TSI, Tuscany in Italy (1KGP); IBS, Iberian populations in Spain (1KGP); FIN-Finnish European (gnomAD); NFE-non-Finnish European, including Northwestern European, Bulgarian, Estonian, Swedish, Southern European, and Other non-Finnish European (gnomAD); AFR, African (gnomAD); EA, East Asian (gnomAD); SA, South Asian (gnomAD); LAT, Latino/Admixed American (gnomAD); ASJ, Ashkenazi Jewish (gnomAD). Barplots are showing frequencies of the alleles associated with lower levels of selected micronutrients. Differences in allelic distributions were analyzed between Serbian and other populations using Chi square test followed by Bonferroni correction for multiple testing. Statistical significance was marked with *(*p* = < 0.008), while statistical trend with #(0.1 < *p* < 0.008).

Finally, we aimed to compare allele frequencies of selected genetic variants in Serbian COVID-19 study group with their frequencies in other populations. Therefore, we retrieved variant site data from the two genetic databases, 1KGP and gnomAD. Distributions of alleles frequencies (AF) associated with decreased levels of selected micronutrients in European and worldwide populations have been presented in Figure 1 (data used for calculations are provided in Supplementary Table 1). For all analyzed genetic variants, observed AF in Serbian study group did not diverge from overall AF in European non-Finish populations. When comparing Serbian study group with populations of non-Finish Europeans, we noticed a difference in the frequency of *DHCR7/NADSYN1* allele G between Italian compared to Serbian population (0.22 and 0.3, respectively, *p* = 0.07). Difference was also observed in the frequency of *CYP2R1* rs10741657 allele G between Serbian (0.58) and Spanish (0.69) as well as Italian population (0.66) (*p* = 0.02, *p* = 0.08, respectively). Serbian population had the lowest frequency of the *CYP2R1* rs10741657 G variant among European non-Finish populations, while Spanish and Italians demonstrated the highest. However, noted differences were not statistically significant. As expected, significant differences were mostly seen between Serbian and African, South and East Asian, American Latino/Admixed, and Ashkenazi populations.

The level of population genetic variability at each selected loci was assessed using delta AF (dAF), representing the difference between maximum and minimum AF value across analyzed populations. The highest dAF was observed for the *DHCR7/NADSYN1* rs12785878 variant (dAF 0.56). The lowest dAF was detected for *CYP2R1* rs10741657 and *DMGDH* rs17823744 variants (dAF 0.15 and 0.15, respectively).

## Discussion

In this study, we focused our attention to genetic variants related to altered level, bioavailability or mechanism of action of vitamin D, zinc and selenium, aiming to investigate their association with different COVID-19 presentation in children and adults. Serbia, like much of Europe and the world, faces micronutrient deficiency/insufficiency which is in part influenced by genetic variability. The prevalence of suboptimal levels of vitamin D and selenium in Serbian population is estimated to be around 50% (32, 33), while zinc insufficiency is around 7% (12). Bearing in mind that even a mild insufficiency of these micronutrients can cause suboptimal immune function, implications for COVID-19 pandemic can be formidable.

Results of our study showed association of *DHCR7/NADSYN1* rs12785878 and *CYP2R1* rs10741657 variants with severe form of COVID-19 in adult patients. In the pediatric COVID-19 group, we did not detect any associations between analyzed genetic factors and disease severity.

Variant rs12785878 is located 8 kilobases upstream of the *DHCR7* gene on chromosome 11q12. *DHCR7* codes for 7-dehydrocholesterol reductase, a key enzyme in cholesterol biosynthesis and vitamin D cutaneous production. Particularly, the activity of DHCR7 enzyme decreases the level of vitamin D precursor availability, 7-dehydorcholesterol, shunting it in the direction of cholesterol biosynthesis (34). *DHCR7* rs12785878 variant was firstly noted in a large GWAS of 25OHD concentrations in 33 996 individuals of European descent by (21), and subsequently confirmed in other numerous studies, where the G allele was shown to be associated with lower 25OHD serum levels. In our comparative genetic population analysis, we showed that frequency of G allele markedly varied across different populations; it ranged from 0.75 in African and Asian populations to 0.22 in European populations. Considerable differences in allele frequencies between populations may indicate a locus that has undergone positive selection in a specific geographical area. It has been suggested that positive selection influenced the *DHCR7* gene, increasing the frequency of reduced activity variant (allele T) in European populations (35). Reduced activity of DHCR7 leads to increased availability of 7-dehydrocholesterol for vitamin D synthesis allowing Europeans to avoid deficiency in northern latitudes. Our results suggested an association of the rs12785878 variant with the severe form of COVID-19. However, in our study, carriers of the TT genotype which has been linked to higher circulating level of 25OHD, were more likely to develop a severe form of COVID-19. Given the immune-enhancing aspects of vitamin D, one might expect the opposite. However, it should be noted that the roll of *DHCR7* variants to vitamin D status is constrained to vitamin D synthesis in the skin exposed to UV light and probably does not influence contribution of dietary sources and supplementation to the level of this vitamin. COVID-19 patients enrolled in our study were hospitalized with this disease in the spring of 2020 during or right after lockdown measures were in place. That means that vitamin D was scarcely synthetized in skin during that time and, therefore, *DHCR7* variants had limited influence on vitamin D level. Regardless of vitamin D production, *DHCR7* variants could influence immune function mediated through cholesterol metabolites. Namely, reduced activity of DHCR7 leads to increased 7-dehydrocholesterol levels which influences interferon production and viral clearance (36).

After initial UV-light mediated production of vitamin D3 in the skin, the second step in the synthesis of active vitamin D is catalyzed in the liver by cytochrome P450 (CYP) enzyme which hydroxylates carbon 25, producing the intermediate 25-hydroxyvitamin D3, or 25OHD, the major circulatory form of the vitamin D (37). This enzyme is encoded by the *CYP2R1* (cytochrome P450, family 2, subfamily R, polypeptide 1) gene. Variant rs10741657 located near the *CYP2R1* gene was linked by several studies to 25OHD serum concentrations (21, 38), with the allele G shown to be associated with lower 25OHD serum levels, and with the GG homozygotes having the lowest levels. Our results indicated an association between severe COVID-19 and *CYP2R1* GG genotype. In the logistic regression model, *CYP2R1* rs10741657 GG carriers had 5.9 times higher odds to develop severe disease. We noticed low level of variability of allele G frequency for *CYP2R1* rs10741657 variant across analyzed populations (dAF 0.15); G allele frequencies ranged from 0.58 in Serbian, which was the lowest among European populations, to 0.73 in African population. The highest frequency of the G allele among Europeans was observed in Italian and Spanish populations (0.66 and 0.69, respectively). Recent study that reported negative correlations between mean levels of vitamin D in various European countries with COVID-19 cases per million people, suggested that Spain, Italy and Switzerland are the most vulnerable countries since they have very low levels of vitamin D in the aging population (39). Our results could be of particular interest for these populations. Relatively high occurrence of the *CYP2R1* rs10741657 G variant in different populations, reproducible association with decreased 25OHD levels and observed relationship with severe COVID-19 in our group of patients, gives a solid ground for future studies to examine relationship of this *CYP2R1* variant with the clinical course of COVID-19 in other populations.

A role of vitamin D in the pathogenesis of COVID-19 has been extensively studied since the beginning of the pandemic. Calcitriol (1, 25-dihydroxyvitamin D3) has an important role in regulating renin angiotensin system by enhancing the expression of ACE2, which is the main target of SARS-CoV2 cell entry (7, 8). Also, vitamin D has an immunomodulatory effect and it can prevent macrophages to release excessive proinflammatory cytokines and chemokines (6, 40). A recent study showed that the intake levels of relevant micronutrients such as vitamin D are inversely associated with higher COVID-19 incidence and/or mortality (41).

Furthermore, other studies sought for more detailed nutrigenetic markers as factors that might contribute to the bioavailability of vitamin D and therefore influence COVID-19 susceptibility and/or clinical course. A study which aimed to assess the association between variants in the *GC* gene that encodes vitamin D binding/transport protein (DBP), and the prevalence and mortality rates of COVID-19, pointed out to rs7041 variant (42). Another recent study (not peer-reviewed yet) performed in Portuguese population of COVID-19 patients found an association of *GC* rs2282679 variant with COVID-19 disease severity (43). Our study indicated an association between *DHCR7/NADSYN1* rs12785878 and *CYP2R1* rs10741657 variants and the severity of COVID-19, but the variants *GC* rs2282679 and *VDR* rs2228570 were not linked to a higher risk of severe COVID-19 in adults. In a recent Mendelian randomization study (not peer-reviewed yet) on vitamin D and COVID-19 in individuals of European ancestry, genetically increased 25OHD concentrations did not protect against COVID-19 susceptibility, but increased the odds for hospitalization and severe disease (44). Another Mendelian randomization study failed to find evidence of a linkage between vitamin D deficiency and COVID-19 infection rates or severe disease (45). However, described studies did not consider true vitamin D deficiency in participants, and it can be possible that vitamin D supplementation could benefit insufficient/deficient patients (44, 45).

Nutrigenetics of zinc and selenium were discussed in the context of COVID-19 as a promising strategy to implement personalized approach to strengthening antioxidant and antiviral defense (41). However, the present study is the first study on genetic determinants of zinc and selenium that included COVID-19 patients. Our results did not show association of these variants with severe COVID-19, however, our univariate analysis pointed out to *DMGDH* rs17823744 variant, which warrants further investigation. DMGDH is implicated in homocysteine metabolism and it is postulated that there is a connection between selenium exposure and the homocysteine metabolic pathways (28). Apart from locus near *DMGDH* gene, which was noted in several GWAS cohorts focused on selenium status, other studies analyzed genes coding for selenoproteins. Candidate gene approach studies or studies that analyzed protein activity, associated glutathione peroxidase 1 (*GPX1*), *GPX4* and selenoprotein P (*SELENOP*) with response to selenium supplementation (46–48). Although important for selenium action, these genes were not associated with selenium status in GWAS studies.

Apart from nutrigenetics, other studies also dealt with genetic factors associated with COVID-19 severity, but the results were not consistent. A GWAS study of almost 4,000 healthy volunteers and severely ill Italian and Spanish COVID-19 patients associated loci on chromosomes 3 and chromosome 9 (near ABO locus) (3), while another GWAS study of 2,244 critically ill UK patients associated variants located on chromosomes 12, 19 and 21 with more severe disease or poor outcome (4). None of these loci are located near nutrigenetic variants analyzed in the present study.

Results of this study should be evaluated in larger cohorts of COVID-19 patients. Small number of patients is the main limitation of this study, although, both mild and moderate, as well as severe groups are well-represented among adult patients. Among pediatric patients, the majority of patients had mild symptoms so we could not investigate risk factors of severe disease in children. Instead, genetic variants were analyzed as risk factors of symptomatic disease.

Another limitation of this study is lack of micronutrient measurement needed to assess actual status of vitamin D, zinc and selenium. We focused solely on the genetic variants associated with altered levels of vitamin D, zinc and selenium. Status of the micronutrient is highly confounded variable that depends on numerous factors. Beside genetics, nutrition habits, individual lifestyle and presence of comorbidities are also important. Therefore, comprehensive nutrigenetic studies complemented with measures of nutritional intake, micronutrient's serum level and detailed lifestyle evaluations are needed in the future.

This study highlights the importance of personalized as well as population based strategies directed toward reduction of COVID-19 burden. These strategies can be implemented through employment of established nutrigenetic markers, such as genetic variants involved in vitamin D disposition. Our results pointed out to *DHCR7/NADSYN1* rs12785878 and *CYP2R1* rs10741657 variants, both involved in vitamin D synthesis, as potential risk factors of severe COVID-19. Holistic approach comprising nutrigenetics, micronutrient, status, lifestyle and clinical parameters could effectively combat micronutrient deficiency, and potentially improve anti-viral defense. This approach is especially important in populations at risk of micronutrient deficiency.

## Data Availability Statement

The raw data supporting the conclusions of this article will be made available by the authors, without undue reservation.

## Ethics Statement

This study was approved by the Ethics Committee of the Institute of Molecular Genetics and Genetic Engineering University of Belgrade (approval for sample collection and biobank formation O-EO-016/2020, 06.05.2020.; approval for the genetic study O-EO-016/2020/1, 03.09.2020).

## Author Contributions

NK: conceptualization, investigation, statistical analysis, writing - draft preparation, and editing. AS: methodology, investigation, and writing – draft preparation. KK: investigation, methodology, and writing – draft preparation. VG: data analysis and interpretation, investigation, and writing – draft preparation. BZ: methodology, investigation, writing — review, and editing, VS-T, MS, ZZ, OO, GS, and LL: methodology, sample collection, and clinical data analysis. SP: concept and design of the study, writing –review, and editing, BS: concept and design of the study, statistical analysis, results interpretation, drafting, and review of final manuscript. All authors contributed to the article and approved the submitted version.

## Conflict of Interest

The authors declare that the research was conducted in the absence of any commercial or financial relationships that could be construed as a potential conflict of interest.
